# Dr. Grenville on Rubia Tinctorum

**Published:** 1811-08

**Authors:** A. B. Grenville

**Affiliations:** Surgeon R. N. Upper Newington, Liverpool


### *To the Editors of the Medical and Physical Journal.*

GENTLEMEN,

IN looking over the 8th volume of your most valuable repository for medical information, I accidentally met with a *notice* of a preparation of the extract of *Rubia tinctorum*, and thus, with no less surprize than pleasure, learned, that Dr. Home had in some of his publications recommended the use of that most valuable root in *Chlorosis*, heedless of the imposing authority of Cullen, who, in his Materia Medica, had recorded it as a nugatory medicine. To account in some manner for my ignorance of this fact, and for the presumption of making remarks upon it, at so remote a period, it may not be impertinent to observe that, although in my *fifth* year of English *denization*, yet from the public situation I embraced on my arrival in this country, and being afterwards constantly on active service abroad, I had few opportunities of gaining periodical information. Having, by the repeated observations of the best practitioners in different parts of Europe, acquired sufficient authority to differ *once* from the opinion of a great master, I embrace the first opportunity of relating to the medical world what I know, of sufficient authenticity, respecting the use and virtues of the *Rubia tinctorum*.

All professional persons that have not forgotten their early *school books* are aware, that this root is recommended by the most celebrated of the Greek physicians under the name of Ερυθροδάνον, and by those of Italy, under that of *Rubia silvestris aspera*, or *Rubia tinctorum sativa*. The virtues which have been ascribed to it, by those writers, have made them agree in its application as an emmenagogue; why

 therefore therefore Cullen, on the simple authority of his own practice, or that of a few friends, chiefly confined to the same spot, should have not only ridiculed the belief in the virtues of that root, but even doubted the veracity of those men of eminence, spread over the most civilized parts of the world, who, convinced by satisfactory experiments, had subscribed to it, and sanctioned its use in medicine, is not easy to conjecture, without bringing in question the modesty of that celebrated man. The fact is, that all those who, after the publication of his Materia Medica, still continued to place faith in the medicinal qualities of the *Rubia*, had reason to applaud themselves for disregarding *his injunctions*. To my own knowledge, the *Rubia tinctorum* has been generally prescribed in France, Italy, and Spain, to this day, in cases of *Chlorosis*, and simple suppression of the *Catamenia;* and to assert with what success, I need only mention that the prescriptions were sanctioned with the names of *Pacchioni*, *Vacca*, *Moschati*, *Bichat*, *Pinel*, *Arejulade*. These highly esteemed physicians have invariably administered the *Rubia tinctorum*, differently prepared, when called upon to correct the deficiency of menstrual evacuations, and I have been more than once a witness to its almost instantaneous good effects.

The *Rubia*, as a remedy possessing high *ecphractic* qualities, can boast of being more *popularly* known than any other article in the Materia Medica; for it is the remedy which is invariably recommended to young chlorotic patients by *old women* in Greece, Turkey, Egypt, Italy, France, Spain, &c. in which countries I found it in the hands of almost every person, who had either suffered or was actually suffering from a suppression of the menstrua. Had not Dr. Cullen disdained to make inquiry "*inter miseram et jejunam plebem* of his own country, and had he possessed patience enough to listen to their tales, and to examine the truth of their assertions, he would, most probably, have classed the *said medicine* with the *few* to whose virtues he yielded belief.

Since my late return from abroad, mingling with the most numerous and the most useful, though the most neglected classes of the inhabitants of a very populous town in England, I have seen two decided cases of *Chlorosis*, and one of *Dysmenorrhœa*, that had baffled the skill of regular practitioners, yielding, and I must repeat it, *instantaneously*, to the *unqualified* prescriptions of "*gossipping neighbours*," consisting in generous doses of *Rubia tinctorum* grossly pulverized. A case of total suppression of the *Catamenia* in a higher rank of life, I have also seen since cured rapidly with the same medicine. I purpose in future to study most particularly its application, and should I have reason, of which which I doubt not, to be confirmed in my opinion as to its efficacy, I shall not fail to communicate to you such cases, as may tend (through your extensive channel of medical information) to restore to its wonted rank a medicine, too improperly neglected.

The powder, in general, is to be preferred, and this may be obtained nearly impalpable. The tincture is equally valid, where the powder is found inconvenient, and the extract after this.

I shall conclude with relating the particulars of the last case above alluded to. Mrs. ———, a young married lady of a most delicate constitution, with light hair, and of a placid temperament, had waited in vain for the space of three months, immediately after the delivery of her first child, for the re-appearance of the *Catamenia.* Already did she feel the bad effects of the obstruction; but not till acute pains in the *loins* and the *hypogastric* region, with a sense of weight within the vagina, swelled ankles, pale face, head-ache, and loss of appetite, rendered her life precarious, were physicians called in for assistance. Warm baths, venesection of the foot, rhubarb, steel, were alternately employed, and with unremitted assiduity, but without success. The case was one day variously deplored by the surrounding circle of female acquaintances, when a certain "*excellent stuff*" was offered, for the cure, by one of the ladies, who had herself experienced *most wonderful* effects from its use in a similar case. Indifferent to any description of medicines, the too much exhausted patient readily submitted to the trial, and within that week she had cause to thank her friend, and to hail the return of health, by the appearance of the menstrual evacuations, which have been marked ever since by the greatest regularity. Of this "*excellent stuff*" I had the curiosity to examine a remaining part, and found it to be the *Rubia tinctorum* in powder, which the lady had procured under the name of *Dyers madder.* Mrs. ——— has since assured me, that on again examining the powder, she is certain of its being similar to what she had taken on a former occasion, when the *first* appearance of the *Catamenia* in her was attended by severe pains, and other distressing symptoms, in which case also the said medicine proved of the greatest advantage*.

A. B. GRENVILLE, M. D.

Surgeon R. N.

*Upper Newington, Liverpool.*

---

* We are obliged to Dr. Grenville for calling the attention of the profession to this neglected remedy, from which, in our own practice, we have occasionally seen considerable benefit derived in cases of amenorrhœa, that had resisted steel and the usual emmenagogues. Further communications on this subject are earnestly requested.

EDITORS.

